# Multivalent adaptor proteins specifically target NK cells carrying a universal chimeric antigen receptor to ErbB2 (HER2)-expressing cancers

**DOI:** 10.1007/s00262-023-03374-x

**Published:** 2023-01-23

**Authors:** Jordi Pfeifer Serrahima, Congcong Zhang, Pranav Oberoi, Malena Bodden, Jasmin Röder, Claudia Arndt, Anja Feldmann, Anne Kiefer, Maren Prüfer, Ines Kühnel, Torsten Tonn, Michael Bachmann, Winfried S. Wels

**Affiliations:** 1 Georg-Speyer-Haus, Institute for Tumor Biology and Experimental Therapy, Paul-Ehrlich-Straße 42-44, 60596 Frankfurt, Germany; 2 Partner Site Frankfurt/Mainz, German Cancer Consortium (DKTK), Frankfurt, Germany; 3 German Cancer Research Center (DKFZ), Heidelberg, Germany; 4 Frankfurt Cancer Institute, Goethe University, Frankfurt, Germany; 5 Institute of Radiopharmaceutical Cancer Research, Helmholtz-Zentrum Dresden-Rossendorf (HZDR), Dresden, Germany; 6 Experimental Transfusion Medicine, Faculty of Medicine Carl Gustav Carus, TU Dresden, Dresden, Germany; 7 Partner Site Dresden, German Cancer Consortium (DKTK), Dresden, Germany; 8 National Center for Tumor Diseases (NCT) and Tumor Immunology, University Cancer Center (UCC) Carl Gustav Carus, TU Dresden, Dresden, Germany; 9 Institute for Transfusion Medicine, German Red Cross Blood Donation Service North-East, Dresden, Germany

**Keywords:** Natural killer cells, NK-92, Chimeric antigen receptor, UniCAR, ErbB2, HER2

## Abstract

Chimeric antigen receptor (CAR)-engineered immune effector cells constitute a promising approach for adoptive cancer immunotherapy. Nevertheless, on-target/off-tumor toxicity and immune escape due to antigen loss represent considerable challenges. These may be overcome by adaptor CARs that are selectively triggered by bispecific molecules that crosslink the CAR with a tumor-associated surface antigen. Here, we generated NK cells carrying a first- or second-generation universal CAR (UniCAR) and redirected them to tumor cells with so-called target modules (TMs) which harbor an ErbB2 (HER2)-specific antibody domain for target cell binding and the E5B9 peptide recognized by the UniCAR. To investigate differential effects of the protein design on activity, we developed homodimeric TMs with one, two or three E5B9 peptides per monomer, and binding domains either directly linked or separated by an IgG4 Fc domain. The adaptor molecules were expressed as secreted proteins in Expi293F cells, purified from culture supernatants and their bispecific binding to UniCAR and ErbB2 was confirmed by flow cytometry. In cell killing experiments, all tested TMs redirected NK cell cytotoxicity selectively to ErbB2-positive tumor cells. Nevertheless, we found considerable differences in the extent of specific cell killing depending on TM design and CAR composition, with adaptor proteins carrying two or three E5B9 epitopes being more effective when combined with NK cells expressing the first-generation UniCAR, while the second-generation UniCAR was more active in the presence of TMs with one E5B9 sequence. These results may have important implications for the further development of optimized UniCAR and target module combinations for cancer immunotherapy.

**Supplementary Information:**

The online version contains supplementary material available at 10.1007/s00262-023-03374-x.

## Introduction

Cytotoxic lymphocytes engineered to express chimeric antigen receptors (CARs) constitute a powerful strategy for adoptive cancer immunotherapy with effector cells of defined target specificity [1–3]. CAR-T cells redirected to the differentiation antigens CD19 or B-cell maturation antigen (BCMA) are in clinical practice for the treatment of various B-cell malignancies, preventing disease recurrence and extending survival for many patients [4]. Nevertheless, the current dependence on autologous, patient-derived T cells can result in clinical products of varying quality and activity, with potentially life-threatening side effects, on-target/off-tumor toxicity and immune escape due to antigen loss representing additional challenges [5–7].

Many of these limitations may be overcome by modular adaptor CAR systems which allow tight control of magnitude and kinetics of target recognition and effector cell activation [8–10]. Such systems consist of two components, a defined CAR that recognizes a hapten or a peptide epitope not naturally exposed on the surface of potential target cells, and a versatile adaptor molecule with tumor-cell specificity that is recognized by the CAR and bridges target and effector cells, thereby inducing CAR activation and target cell lysis [11, 12]. The respective UniCAR approach is based on a universal CAR which recognizes a linear epitope (E5B9) of the nuclear autoantigen La/SS-B, and bispecific adaptor proteins termed target modules (TMs) that harbor a tumor-specific scFv antibody domain fused to the E5B9 peptide [13]. Activation of UniCAR-T cells is strictly dependent on the crosslinking of effector and target cells by a suitable TM or TM combination, preventing uncontrolled CAR-T cell expansion and allowing subsequent or simultaneous targeting of a second tumor antigen to counteract immune escape [12]. A recent proof-of-concept study in patients with relapsed or refractory acute myeloid leukemia demonstrated clinical utility of this approach, with UniCAR-engineered autologous T cells combined with a CD123-specific TM resulting in clinical responses without inducing severe side effects [14].

Natural killer (NK) cells constitute a promising alternative lymphocyte population for CAR engineering [15–17]. In contrast to T cells, NK cells do not carry a T-cell receptor, markedly reducing the risk of graft-versus-host disease even if applied as an HLA-unmatched allogeneic cell therapeutic [18]. Consequently, expression of an adaptor CAR such as the UniCAR in NK cells may lead to true off-the-shelf therapeutics, with the same engineered cell product suitable for the treatment of different patients, but specific tumor-targeting provided by a recombinant target module chosen according to disease indication and individual tumor characteristics. Based on the continuously expanding and clinically usable human NK cell line NK-92, we previously validated this concept by demonstrating specific cytotoxicity of UniCAR-NK cells toward tumor cells expressing the disialoganglioside GD_2_ in the presence of GD_2_-specific TMs [19].

Here, we extended this approach to target UniCAR-NK-92 cells to cancer cells of solid tumor origin overexpressing the tumor-associated antigen ErbB2 (HER2). To investigate the effects of increased valency of respective target modules and the distance between UniCAR and tumor-cell binding domains on UniCAR activity, we developed different homodimeric TM designs with one, two or three E5B9 peptides per TM monomer, with the ErbB2-specific scFv antibody fragment and UniCAR epitopes either directly linked or separated by an IgG4 Fc domain. As a prerequisite for further development of optimized UniCAR and TM combinations, we investigated functionality of the recombinant target modules in comparative binding studies and cell killing experiments with ErbB2-positive cancer cells of different solid tumor origins and NK cells carrying a first- or second-generation UniCAR.

## Materials and methods

### Cells and culture conditions

MDA-MB-453, MDA-MB-468 and JIMT-1 breast carcinoma, LN-229 glioblastoma, SK-OV-3 ovarian carcinoma, HEK 293 T embryonic kidney (all ATCC, Manassas, VA) and MZ-Mel-2 melanoma cells (kindly provided by Elke Jäger, Krankenhaus Nordwest, Frankfurt, Germany) were cultured in DMEM medium (Gibco, Thermo Fisher Scientific, Darmstadt, Germany), SK-BR-3 breast carcinoma cells (ATCC) in Advanced DMEM/F12 medium (Gibco, Thermo Fisher Scientific), and K562 erythroleukemia cells (ATCC) in RPMI 1640 medium (Gibco, Thermo Fisher Scientific). All media were supplemented with 10% heat-inactivated FBS (Capricorn Scientific, Ebsdorfergrund, Germany), 2 mM L-glutamine, 100 U/mL penicillin and 100 µg/mL streptomycin (all Gibco, Thermo Fisher Scientific). Expi293F cells were cultured in Expi293 expression medium (both Gibco, Thermo Fisher Scientific). NK-92 cells [20] (kindly provided by NantKwest, Inc., Culver City, CA) and ErbB2-specific NK-92/5.28.z cells [21, 22] were cultured in X-VIVO 10 medium (Lonza, Cologne, Germany) supplemented with 5% heat-inactivated human AB plasma (German Red Cross Blood Donation Service Baden-Württemberg-Hessen, Frankfurt, Germany) and 100 IU/mL IL-2 (Proleukin; Novartis Pharma, Nürnberg, Germany).

### Generation of UniCAR-expressing NK cells

**Fig. 1:**
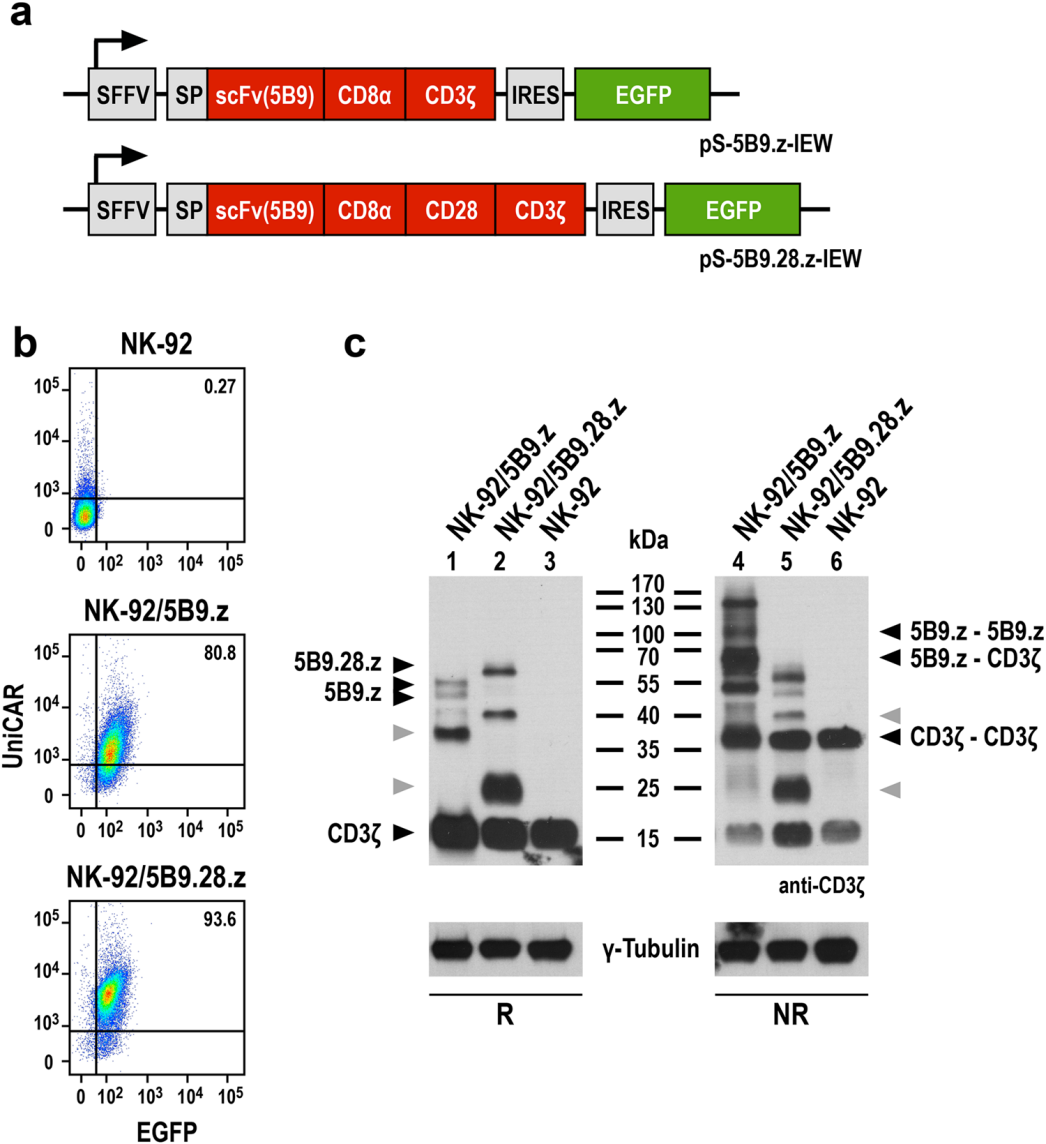
Generation of UniCAR-NK-92 cells. **a** Lentiviral transfer plasmids encoding first- and second-generation UniCARs under the control of the Spleen Focus Forming Virus promoter (SFFV). In pS-5B9.z-IEW the chimeric antigen receptor consists of an immunoglobulin heavy-chain signal peptide (SP), the 5B9 scFv antibody fragment specific for the E5B9 epitope, a CD8*α* hinge region (CD8*α*), and transmembrane and intracellular domains of CD3*ζ* (first-generation UniCAR 5B9.z). The pS-5B9.28.z-IEW vector encodes a similar CAR, but with transmembrane and intracellular domains of the costimulatory CD28 molecule and the intracellular domain of CD3*ζ* (second-generation UniCAR 5B9.28.z). The CAR sequences are followed by an internal ribosome entry site (IRES) and enhanced green fluorescent protein (EGFP) cDNA. **b** EGFP expression of sorted NK-92/5B9.z and NK-92/5B9.28.z cells was determined by direct flow cytometry. UniCAR surface expression was detected using His-Tag-conjugated Protein L and His-Tag-specific secondary antibody. Parental NK-92 cells served as control. **c** Lysates of UniCAR-engineered and parental NK-92 cells were subjected to SDS-PAGE under reducing (R, left panel) or non-reducing conditions (NR, right panel) and subsequent immunoblotting with CD3*ζ*-specific antibody. The positions of CAR monomers, CAR 5B9.z homodimers, heterodimers of CAR 5B9.z with endogenous CD3*ζ*, and CD3*ζ* homodimers are indicated by black arrowheads. Bands likely representing products of proteolytic degradation are indicated by gray arrowheads. γ-Tubulin served as loading control

Universal chimeric antigen receptor (UniCAR) sequences were designed by in silico assembly of an immunoglobulin heavy-chain signal peptide, an extracellular scFv antibody domain derived from antibody 5B9 specific for the E5B9 epitope [13], a modified CD8*α* hinge region [21], and either the transmembrane and intracellular domains of CD3*ζ* (UniprotKB: P20963-3, amino acid residues 29–163) (UniCAR 5B9.z), or the transmembrane and intracellular domains of CD28 (UniprotKB: P10747, amino acid residues 151–220) linked to the intracellular domain of CD3*ζ* (UniprotKB: P20963-3, amino acid residues 52–163) (UniCAR 5B9.28.z) (Fig. 1a). Codon-optimized UniCAR sequences were de novo synthesized (GeneArt, Thermo Fisher Scientific) and inserted into lentiviral transfer plasmid pHR’SIN-cPPT-SIEW upstream of IRES and enhanced green fluorescent protein (EGFP) sequences [23], resulting in the vectors pS-5B9.z-IEW and pS-5B9.28.z-IEW. VSV-G pseudotyped vector particles were produced in HEK 293 T cells, and NK-92 cells were transduced as previously described [21]. Transduced NK-92 cells were enriched by sorting EGFP-positive cells with a FACSAria Fusion Flow Cytometer (BD Biosciences, Heidelberg, Germany). UniCAR expression was confirmed by flow cytometry as described below and by SDS-PAGE of cell lysates and immunoblot analysis with antihuman CD3*ζ* antibody (Santa Cruz Biotechnology, Heidelberg, Germany), followed by HRP-conjugated secondary antibody (Sigma-Aldrich, Taufkirchen, Germany) and chemiluminescent detection.

### Design, expression and purification of recombinant target modules

**Fig. 2:**
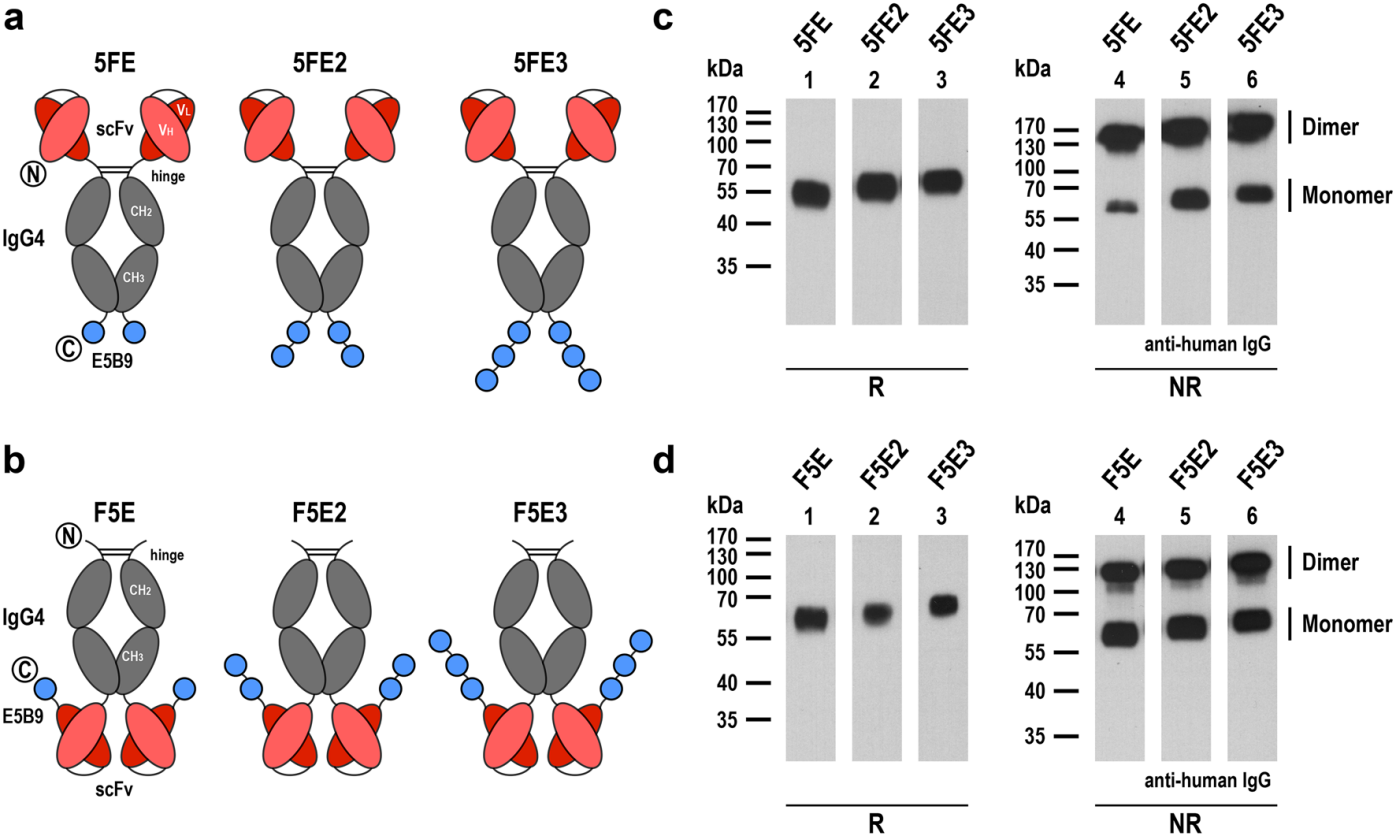
Expression of recombinant target modules. **a** Schematic representation of bispecific target modules 5FE, 5FE2 and 5FE3 consisting of an N-terminal ErbB2-specific scFv antibody fragment, hinge, CH2 and CH3 domains of human IgG4, followed by one, two or three E5B9 peptide sequences per monomer separated by G_4_S linkers. Amino (N) and carboxy termini (C) of the proteins are indicated. Disulfide bridges within the hinge region facilitate homodimerization. **b** In target modules F5E, F5E2 and F5E3 the ErbB2-specific antibody domain is placed C-terminal of the IgG4 Fc region, followed by the E5B9 peptide sequences. **c, d** Immunoblot analysis of purified target modules under reducing (R) or non-reducing (NR) conditions with HRP-conjugated antihuman IgG antibody. The positions of TM monomers and dimers are indicated

Sequences encoding the IgG4-based target modules 5FE, 5FE2 and 5FE3 were designed by in silico assembly of an immunoglobulin heavy-chain signal peptide, an scFv antibody domain derived from ErbB2 (HER2)-specific antibody FRP5 [24], hinge, CH2 and CH3 domains of human IgG4 (UniprotKB: P01861, amino acid residues 104–327), a G_4_S-linker, the E5B9 epitope recognized by the UniCARs (5FE) [13], or two or three repeats thereof, each separated by a G_4_S-linker (5FE2 and 5FE3) (Fig. 2a). Alternatively, the scFv(FRP5) domain was placed C-terminal of the IgG4 Fc domain, connected via a G_4_S-linker to one, two or three E5B9 sequences, each again separated by a G_4_S-linker, resulting in TMs F5E, F5E2 and F5E3 (Fig. 2b). Codon-optimized sequences were de novo synthesized (GeneArt, Thermo Fisher Scientific) and inserted into mammalian expression vector pcDNA3. Expi293F cells were transiently transfected with the resulting plasmids using the ExpiFectamine 293 transfection kit following the manufacturer's recommendations (Gibco, Thermo Fisher Scientific), and recombinant proteins were purified from culture supernatants by affinity chromatography using a Protein G column (Pierce, Thermo Fisher Scientific) on an ÄKTA FPLC system (GE Healthcare Europe, Freiburg, Germany). Purity and identity of the isolated TMs were confirmed by SDS-PAGE, either followed by Coomassie staining or immunoblotting with HRP-conjugated antihuman IgG antibody (Sigma-Aldrich) and chemiluminescent detection. Protein concentrations were determined using a Nanodrop 1000 spectrophotometer with the calculated molecular mass and extinction coefficients (Thermo Fisher Scientific). Protein bands in immunoblots were quantified by densitometry using ImageJ 1.53 software (https://imagej.net).

### Flow cytometric analysis

ErbB2 surface expression on tumor cells was determined using an APC-conjugated ErbB2-specific antibody (BioLegend, Koblenz, Germany). Expression of first- and second-generation UniCARs on the surface of transduced NK-92 cells was analyzed with His-Tag-conjugated Protein L (AcroBiosystems, Newark, NJ), followed by APC-conjugated His-Tag-specific secondary antibody (BioLegend). Binding of purified TMs to UniCAR-NK or tumor cells was investigated by incubating the cells with 12.5 nM of the respective protein, followed by staining with an APC-conjugated antihuman IgG secondary antibody (Jackson ImmunoResearch, Cambridgeshire, UK). All samples were then analyzed with a FACSCanto II flow cytometer (BD Biosystems). Data were evaluated using FlowJo software (Version 10.6.2; FlowJo, Ashland, OR). The relative binding strength of different TMs to UniCARs was assessed in a competitive binding assay. Purified 5FE protein was covalently labeled with APC (Lightning-Link; Abcam, Cambridge, UK) following the manufacturer's recommendations. Then UniCAR-NK-92 cells were incubated with 9.52 nM of APC-conjugated 5FE in the presence of increasing concentrations of 0.019 to 47.57 nM of a second unlabeled ErbB2-specific TM or a PD-L1-specific IgG4 Fc protein as an isotype control (kindly provided by Aline Häcker, Georg-Speyer-Haus). After 2 h, cells were washed with PBS containing 1% FBS, and remaining APC-conjugated 5FE still bound to the UniCAR-NK-92 cells was determined using an LSRFortessa Cell Analyzer (BD Biosciences).

### Cytotoxicity assays

Tumor cells were stained with calcein violet AM (CV) (CellTrace; Invitrogen, Thermo Fisher Scientific) and incubated with UniCAR-expressing or parental NK-92 cells at an effector to target (E/T) ratio of 5:1 for 3 h at 37 °C in the absence or presence of 0.64 nM of a target module. After centrifugation and removal of supernatant, cell pellets were resuspended in 80 µl of a 1 µg/mL propidium iodide (PI) solution and analyzed using a FACSCanto II Flow Cytometer. Target cells incubated without effector cells served as control for spontaneous lysis. Dead target cells were identified as CV and PI double positive. Spontaneous target cell lysis was subtracted to calculate specific cytotoxicity. Data were analyzed using FACSDiva software (BD Biosciences).

### Statistical analysis

Quantitative data are expressed as mean and standard deviation (SD). Statistical significance was determined using paired Student's *t*-test. *P* values < 0.05 were considered statistically significant. All analyses were performed using Prism 9 software (GraphPad Software, San Diego, CA).

## Results

### Expression of universal chimeric antigen receptors in NK cells

For comparative analysis, we generated first- and second-generation UniCARs, both encompassing an N-terminal scFv antibody fragment specific for the E5B9 peptide epitope of the nuclear autoantigen La/SS-B [13], fused via a CD8*α* hinge region either to transmembrane and intracellular domains of CD3*ζ* (UniCAR 5B9.z), or transmembrane and intracellular domains of the costimulatory CD28 molecule and CD3*ζ* intracellular domain (UniCAR 5B9.28.z) (Fig. 1a). UniCAR-NK cells were derived by transduction of the clinically applicable human NK cell line NK-92 with VSV-G pseudotyped lentiviral vectors encoding the 5B9.z or 5B9.28.z sequences together with enhanced green fluorescent protein as a marker. Transduced UniCAR-NK-92 cells were enriched by flow cytometric cell sorting. Identity of the selected cell populations and comparable surface expression of UniCARs 5B9.z and 5B9.28.z was confirmed by flow cytometry, analyzing EGFP signals and simultaneously detecting the CARs with Protein L that binds to the κ light chain sequence of the E5B9-specific scFv antibody fragment [25] (Fig. 1b). To assess potential dimer formation, UniCAR protein expression was further examined by immunoblot analysis of whole cell lysates from NK-92 cells carrying the 5B9.z or 5B9.28.z constructs. Under reducing conditions, both UniCAR molecules were detected as monomers with a molecular mass close to the expected values of 48.4 (5B9.z) and 54 kDa (5B9.28.z) (Fig. 1c, left panel). Thereby, CAR 5B9.z appeared as a double band likely reflecting differential glycosylation. Under non-reducing conditions, additional bands were observed in lysates of UniCAR-NK-92 cells harboring 5B9.z, representing UniCAR homodimers and heterodimers of this chimeric receptor with endogenous CD3*ζ*. This was not the case for lysates of UniCAR-NK-92 cells carrying 5B9.28.z, indicating that the latter cannot form covalently linked dimers (Fig. 1c, right panel).

### Generation of multivalent target modules binding to UniCAR and ErbB2

To specifically direct UniCAR-NK cells to the tumor-associated antigen ErbB2, we designed a target module termed 5FE, which reflects structure and molecular mass of an IgG molecule and encompasses an N-terminal scFv antibody domain derived from ErbB2-specific antibody FRP5 [24], linked via hinge, CH2 and CH3 domains of the human IgG4 Fc portion to a C-terminal E5B9 epitope (Fig. 2a, left). Thereby, the Fc domain can provide flexibility to the molecule and enable dimerization via its disulfide bridges [19, 26]. To analyze the possible contribution of avidity effects to the binding of a TM to UniCAR-expressing NK cells, two similar molecules were generated, which either carry two or three E5B9 peptide sequences at their C-terminus (5FE2 and 5FE3; Fig. 2a, middle and right). In prior studies with UniCAR-T cells, monomeric TM molecules were used in which tumor-specific scFv antibody domains were directly fused to the E5B9 epitope [13]. Since crosslinking of target antigen and UniCAR in the immunological synapse may be influenced by the distance between the binding domains of the target module, we also designed three additional fusion proteins termed F5E, F5E2 and F5E3, which carry the IgG4 Fc domain at the N-terminus, followed by the FRP5-derived scFv antibody fragment and one, two or three E5B9 epitope sequences (Fig. 2b).

Codon-optimized TM sequences were fused to an immunoglobulin heavy-chain signal peptide in a pcDNA3 expression plasmid. Then, the recombinant molecules were expressed as secreted proteins in transiently transfected Expi293F cells and purified from culture supernatants by Protein G affinity chromatography. SDS-PAGE followed by Coomassie staining or immunoblot analysis with a human IgG-specific antibody confirmed purity and identity of the fusion proteins in the elution fractions (exemplarily shown for TM F5E2 in Supplementary Fig. 1). To assess formation of dimers, the purified molecules were further examined by immunoblot analysis under reducing and non-reducing conditions. Indeed, TMs 5FE, 5FE2 and 5FE3 were mostly produced as tetravalent, disulfide-bridged homodimers, with only a small proportion of monomers present (Fig. 2c), while in the case of F5E, F5E2 and F5E3 the purified proteins contained similar amounts of homodimers and monomers (Fig. 2d).

### Bispecific binding of target modules to UniCARs and ErbB2

**Fig. 3:**
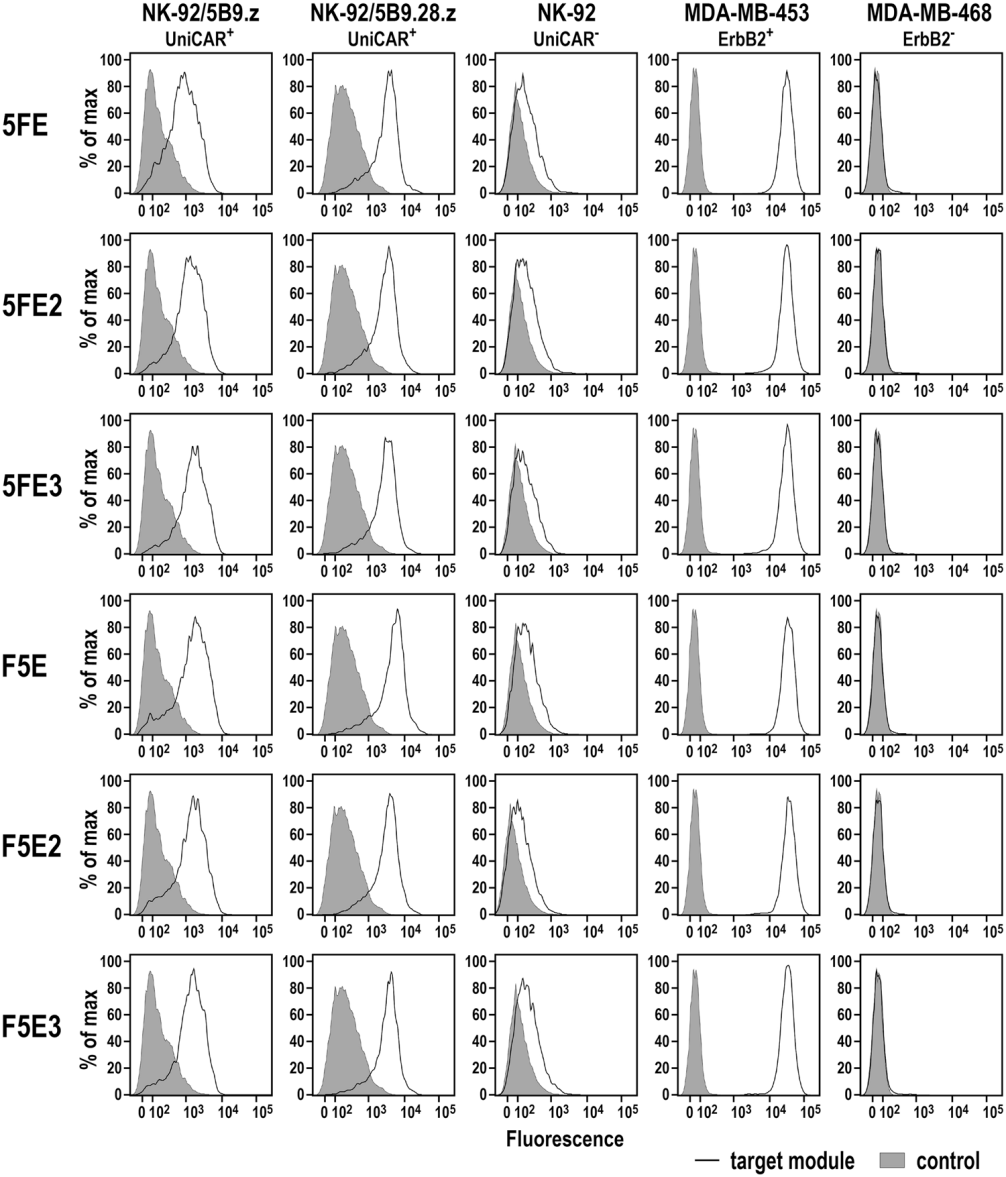
Binding of purified target modules to UniCAR-expressing NK-92/5B9.z and NK-92/5B9.28.z cells, and ErbB2-expressing MDA-MB-453 breast carcinoma cells was investigated by flow cytometry with antihuman IgG antibody (solid lines). Cells stained with detection antibody in the absence of a TM (filled areas), and parental NK-92 cells and ErbB2-negative MDA-MB-468 breast carcinoma cells served as controls

To test binding of the six antibody fusion proteins to ErbB2 via their scFv(FRP5) domain and recognition of the incorporated E5B9 epitope peptides by UniCARs 5B9.z and 5B9.28.z, ErbB2-positive MDA-MB-453 breast carcinoma cells (Supplementary Fig. 2), and UniCAR-expressing NK-92/5B9.z and NK-92/5B9.28.z cells were incubated with the purified target modules, and surface binding was analyzed by flow cytometry. Thereby, 5FE, 5FE2 and 5FE3, and F5E, F5E2 and F5E3 proteins all specifically interacted with ErbB2-positive cancer cells and UniCAR-expressing immune effectors, while no binding to ErbB2-negative MDA-MB-468 breast carcinoma cells used as a control and only background binding to parental NK-92 cells was found (Fig. 3). Likewise, the six recombinant adaptor proteins specifically bound to a panel of additional breast carcinoma, ovarian carcinoma, glioblastoma and melanoma cell lines with high or moderate ErbB2 expression (Supplementary Fig. 3).

**Fig. 4:**
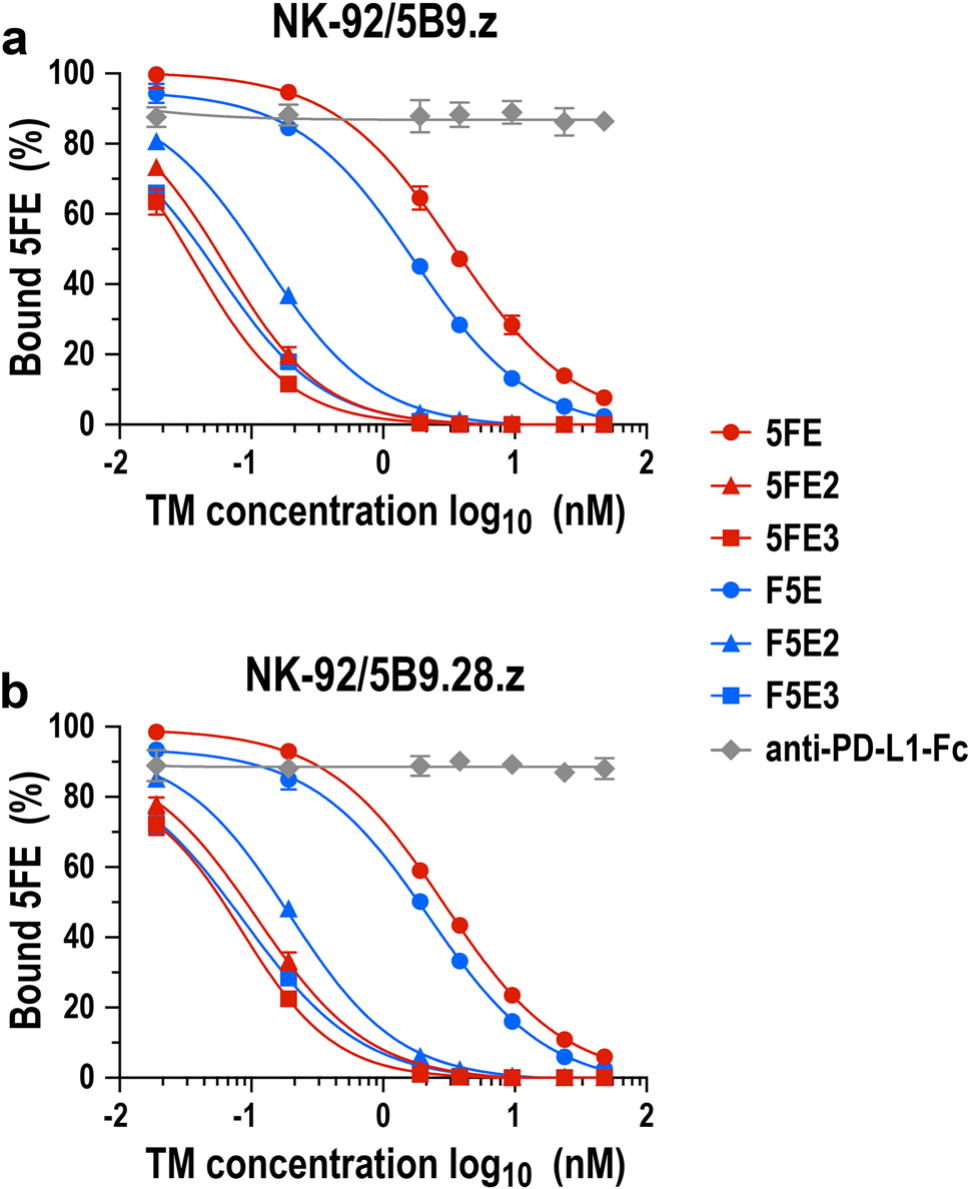
Competitive binding of target modules to UniCAR-NK-92 cells. NK-92/5B9.z (**a**) and NK-92/5B9.28.z cells (**b**) were incubated with 9.52 nM of APC-labeled 5FE protein in the presence of increasing concentrations of unlabeled TM proteins as indicated, washed, and remaining bound APC-5FE was determined by flow cytometry. A PD-L1-specific IgG4 Fc fusion protein served as control. Mean values ± SD from 3 individual experiments and interpolated sigmoidal curves are shown

To quantify potential avidity effects with respect to UniCAR binding contributed by the varying number of E5B9 peptides in the adaptor proteins, competitive binding assays with APC-labeled 5FE and increasing concentrations of unlabeled TMs as competitors were performed, and resulting IC_50_ values were calculated. Thereby, irrespective of whether the IgG4 Fc domain was placed in-between or N-terminal of scFv(FRP5) and E5B9 sequences, TMs containing two or three E5B9 motifs per monomer competed binding of labeled 5FE to UniCAR-expressing cells more effectively than unlabeled 5FE and F5E proteins (Fig. 4). While an increase from two to three E5B9 sequences per monomer still resulted in slightly improved binding and decreased IC_50_ values, the most dramatic differences were observed between 5FE and 5FE2, with a 57-fold and 26-fold lower IC_50_ value for the latter in the case of NK-92/5B9.z or NK-92/5B9.28.z cells, respectively (Table 1). Interestingly, whereas 5FE-binding to the second-generation CAR 5B9.28.z was competed in a similar manner by unlabeled 5FE and F5E, the target module F5E was twice as effective as unlabeled 5FE in competing binding of APC-conjugated 5FE to the first-generation CAR 5B9.z on NK-92 cells. For both UniCAR cell lines, an unrelated PD-L1-specific IgG4 Fc fusion protein included as a control did not affect binding of 5FE, demonstrating specificity of the 5B9/E5B9 interaction. These data show that increasing the number of E5B9 epitopes in 5FE- and F5E-based TMs indeed resulted in enhanced binding to the UniCARs.

**Table 1:** Relative binding strength of ErbB2-specific target modules

	NK-92/5B9.z		NK-92/5B9.28.z	
Target module	IC_50_ (nM)^a^	*R*^2^	IC_50_ (nM)	*R*^2^
5FE	3.44	0.9974	2.89	0.9995
5FE2	0.06	0.9931	0.11	0.9934
5FE3	0.04	0.9926	0.08	0.9753
F5E	1.72	0.9969	2.23	0.9956
F5E2	0.13	0.9952	0.19	0.9961
F5E3	0.06	0.9825	0.09	0.9900

^a^IC_50_ and *R*^2^ values were calculated from the data shown in Fig. 4 by nonlinear regression

### TM-mediated redirection of UniCAR-NK cells

**Fig. 5:**
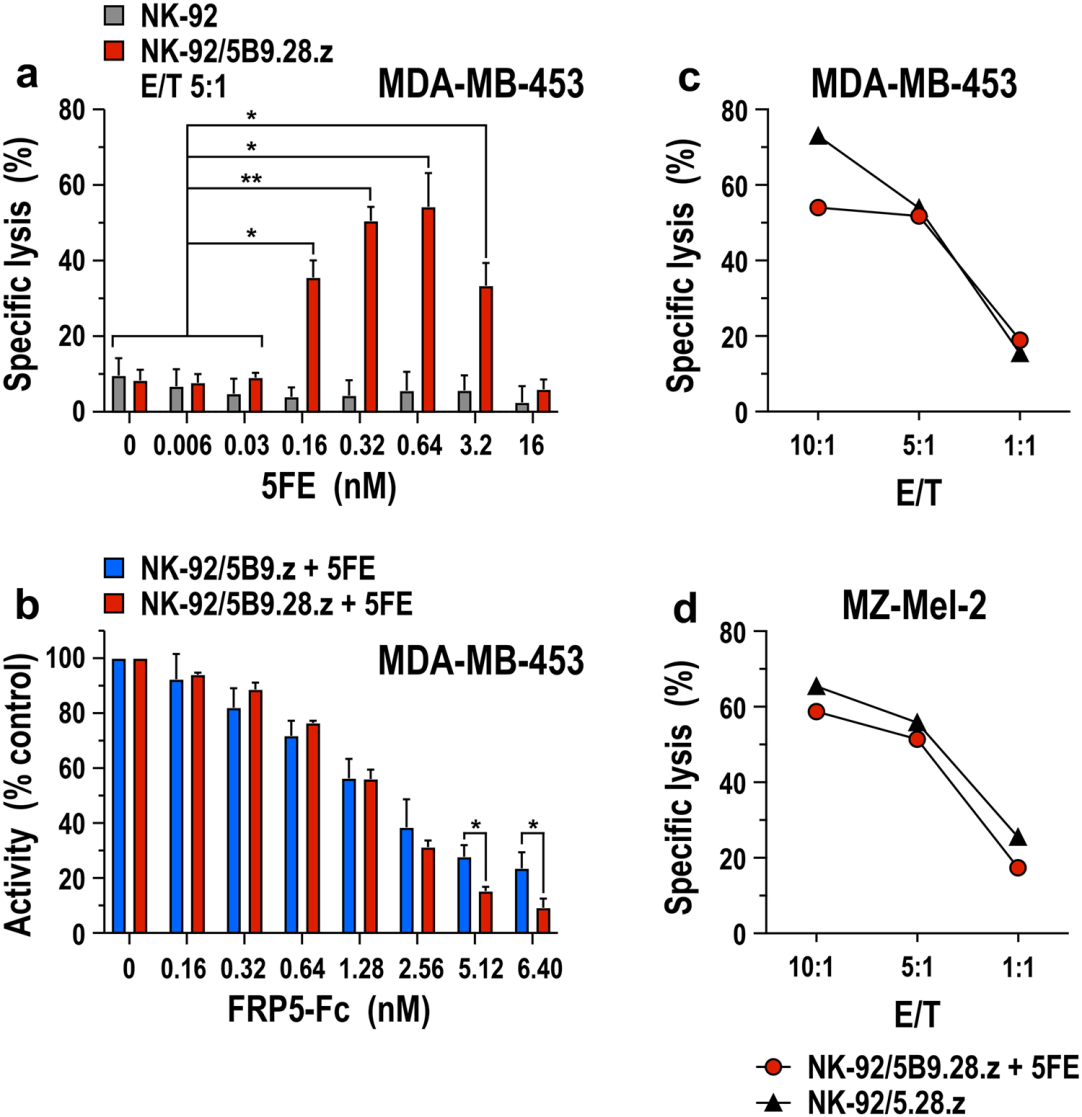
Effect of target modules on the cell killing activity of UniCAR-NK cells. **a** Cytotoxic activity of NK-92/5B9.28.z cells against ErbB2-expressing MDA-MB-453 breast carcinoma cells in the absence or presence of increasing concentrations of purified 5FE target module was investigated in flow cytometry-based cytotoxicity assays after co-incubation at an effector to target ratio (E/T) of 5:1 for 3 h (red bars). Parental NK-92 cells were included as control (gray bars). **b** Specificity of TM-mediated killing by NK-92/5B9.z (blue bars) and NK-92/5B9.28.z UniCAR-NK cells (red bars) was investigated in cytotoxicity assays with MDA-MB-453 target cells at an E/T ratio of 5:1 for 3 h in the presence of 0.64 nM 5FE protein and increasing concentrations of ErbB2-specific FRP5-Fc mini-antibody as a competitor. Mean values ± SD are shown in panels **a**, **b** *n* = 3. * *p* < 0.05, ** *p* < 0.01. **c**, **d** For direct comparison, TM-mediated cytotoxic activity of NK-92/5B9.28.z UniCAR-NK cells and TM-independent cell killing of NK-92/5.28.z cells that express a regular ErbB2-specific CAR against ErbB2-positive MDA-MB-453 breast carcinoma and MZ-Mel-2 melanoma cells was investigated after co-incubation for 3 h at the indicated E/T ratios. MDA-MB-453 cells were pre-incubated with an excess of 5FE protein, washed and then exposed to UniCAR-NK cells, while MZ-Mel-2 cells were kept in the continuous presence of 0.64 nM of 5FE protein. Data from representative experiments are shown

Using the prototypic 5FE molecule together with NK-92/5B9.28.z cells, we first assessed concentration-dependent effects of the target module on specific cytotoxicity of the UniCAR-NK cells toward ErbB2-positive MDA-MB-453 cells. At an effector to target cell (E/T) ratio of 5:1, we observed a steady increase in specific cell killing at 5FE concentrations ranging from 0.006 to 0.64 nM (Fig. 5a). Cytotoxic activity decreased again at higher TM levels, indicative of competition by free TM protein once saturation of UniCAR and/or ErbB2 binding sites was reached. In contrast to NK-92/5B9.28.z cells, 5FE had no effect on cytotoxic activity of parental NK-92 cells. To further confirm specificity of TM-mediated targeting, MDA-MB-453 cells were cocultured with UniCAR-expressing NK-92/5B9.z or NK-92/5B9.28.z cells in the presence of 0.64 nM of purified 5FE protein and increasing concentrations of an FRP5-Fc mini-antibody as a competitor, which consists of the same ErbB2-specific scFv domain used for 5FE, but linked to the Fc portion of human IgG1 without incorporated E5B9 peptide [26]. As expected, cytotoxic activity of both, NK-92/5B9.z and NK-92/5B9.28.z cells decreased gradually with increasing concentrations of the ErbB2-specific competitor, demonstrating strict dependence of the UniCAR-NK cells on TM-mediated recognition of ErbB2 (Fig. 5b). Interestingly, at higher concentrations of the competitor (8- to tenfold over TM), the activity of NK-92 cells expressing the second-generation UniCAR 5B9.28.z was more strongly inhibited than that of the cells harboring the first-generation UniCAR 5B9.z. In contrast to monomeric 5B9.28.z, UniCAR 5B9.z is mostly expressed in the form of 5B9.z/CD3*ζ* heterodimers (see Fig. 1c), which contribute six ITAMs per TM-binding receptor complex that may result in more robust CAR activation even when access to the TM target antigen is limited.

To test the effect of TM-mediated redirection of UniCAR-NK cells in comparison with NK-92 cells carrying a classical CAR, cytotoxic activity of NK-92/5B9.28.z cells in the presence of 5FE was analyzed side by side with that of clinical-grade NK-92/5.28.z cells, which harbor a standard CD28- and CD3*ζ*-based second-generation CAR with the same ErbB2-specific antibody fragment incorporated in the 5FE protein [21, 22]. Thereby, at different E/T ratios and with both, MDA-MB-453 breast carcinoma cells that highly overexpress ErbB2 and MZ-Mel-2 melanoma cells with more moderate ErbB2 expression as targets, comparable cell killing activity of the UniCAR-NK/TM combination and regular CAR-NK cells was found (Fig. 5c, d).

### Differential effects of multivalent target modules on UniCAR-NK cell activity

**Fig. 6:**
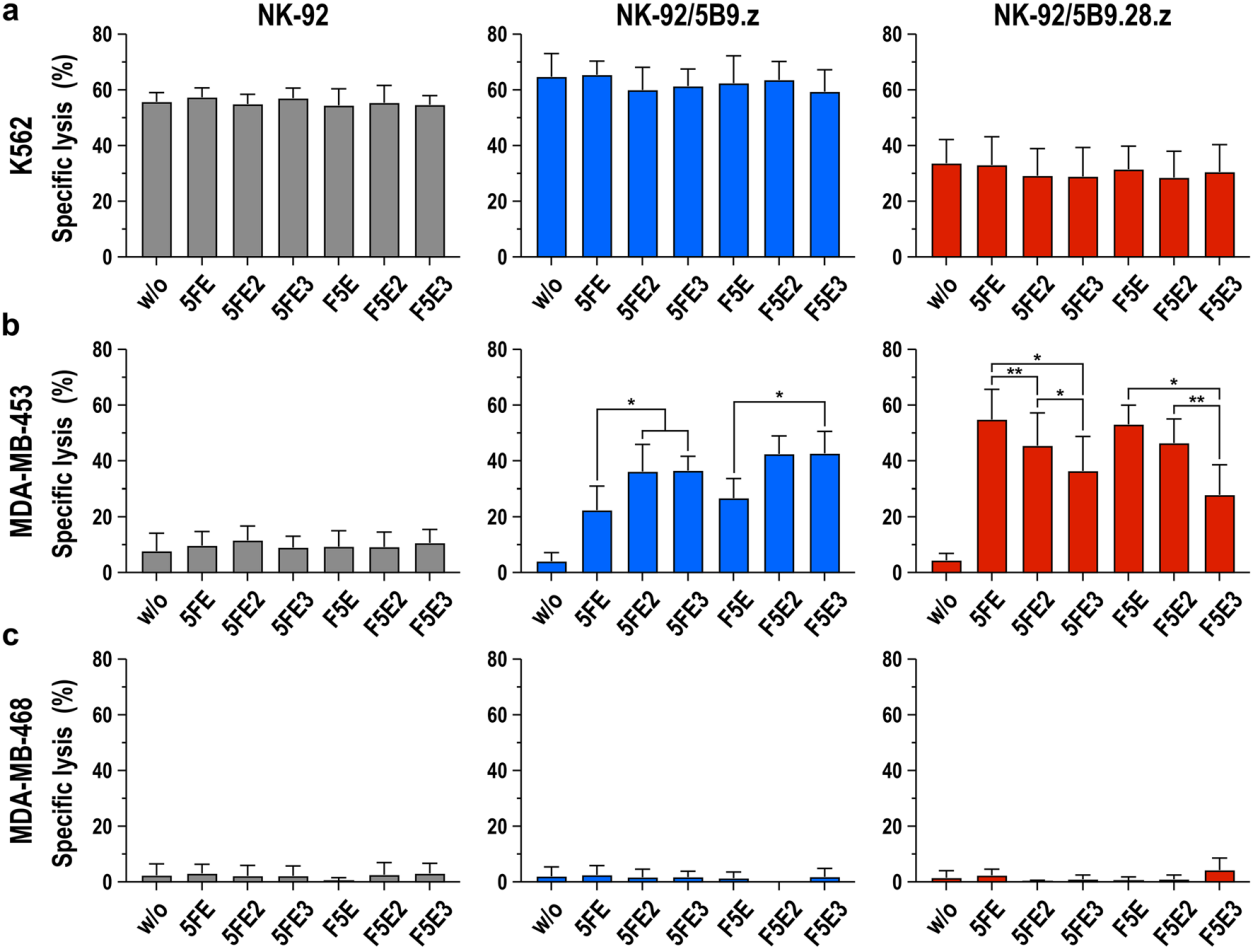
Differential effects of ErbB2-specific target modules on the cytotoxic activity of UniCAR-NK cells. **a** Natural cytotoxicity of NK-92/5B9.z (blue bars) and NK-92/5B9.28.z cells (red bars) against K562 erythroleukemia cells in the absence or presence of 0.64 nM of target modules 5FE, 5FE2, 5FE3, F5E, F5E2 or F5E3 was determined after co-incubation at an E/T ratio of 5:1 for 3 h. Parental NK-92 cells were included for comparison (gray bars). Likewise, specific effects of the different TM proteins on cytotoxic activity of the UniCAR-NK cells against ErbB2-expressing MDA-MB-453 (**b**) and ErbB2-negative MDA-MB-468 breast carcinoma cells (**c**) were investigated. Mean values ± SD are shown; *n* = 3. * *p* < 0.05, ** *p* < 0.01

Next, we evaluated the cytotoxic activity of both, NK-92/5B9.z and NK-92/5B9.28.z cells combined with either one of the six different ErbB2-specific TMs. To assess the possible influence of TMs or UniCAR expression on natural cytotoxicity, MHC-I-negative K562 erythroleukemia cells which are highly sensitive to NK-mediated cytotoxicity but negative for ErbB2 were incubated with the UniCAR-NK cells at an E/T ratio of 5:1 in the absence or presence of 0.64 nM of the target modules. Likewise, TM- and UniCAR-mediated cytotoxicity was analyzed using ErbB2-positive MDA-MB-453 cells as targets. ErbB2-negative MDA-MB-468 cells and parental NK-92 cells served as controls. NK-92 cells expressing the first-generation UniCAR 5B9.z displayed very similar natural cytotoxicity against K562 cells when compared to parental NK-92 cells, which was not affected by the presence of the different TM proteins (Fig. 6a). Interestingly, while also not affected by the target modules, cell killing activity of NK-92/5B9.28.z against K562 cells was reduced in comparison with NK-92 and NK-92/5B9.z cells. We previously observed similar effects with other NK-92 derivatives that carried a CD28-containing CAR [27], possibly due to sequestration of signaling molecules by such CARs away from endogenous CD28 of NK-92 cells which may contribute to natural cytotoxicity against K562 cells [28, 29].

While parental NK-92 cells displayed only low basic activity against ErbB2-positive MDA-MB-453 cells which was independent from the presence of a target module, addition of either one of the TMs significantly increased specific cytotoxicity of NK-92/5B9.z and NK-92/5B9.28.z cells (Fig. 6b). In the case of NK-92 cells expressing CAR 5B9.z, including two or three E5B9 motifs in 5FE- and F5E-based TMs increased cell killing activity to a similar extent, which is in agreement with the findings from the competition binding experiments (see Fig. 4). Unexpectedly, this was different for NK-92/5B9.28.z cells, which were more active against MDA-MB-453 cells in the presence of the target modules 5FE and F5E, and showed a gradual decline in cell killing if two or three E9B9 peptides were included in the recombinant adaptor proteins. While less pronounced, a similar trend was observed for other target tumor cells with high or more moderate ErbB2 expression (Supplementary Fig. 4). Despite lower CAR surface expression (see Fig. 1b), NK-92/5B9.z cells in the presence of TMs 5FE2 or F5E2 were thereby more active against some of the targets than NK-92/5B9.28.z cells combined with TMs 5FE or F5E, most likely due to recruitment of endogenous CD3*ζ* by UniCAR 5B9.z and more pronounced natural cytotoxicity of the former, which may contribute to antitumoral activity. Unlike E5B9 valency, the different spacing of the tumor-specific binding domain and the peptide recognized by the UniCARs in 5FE- and F5E-based TMs had little effect on specific cytotoxicity of NK-92/5B9.z and NK-92/5B9.28.z cells. In the case of NK-resistant and ErbB2-negative MDA-MB-468 control cells, the targets remained unaffected by parental NK-92, NK-92/5B9.z or NK-92/5B9.28.z cells irrespective of the presence of a target module (Fig. 6c). Taken together, these data indicate that all recombinant adaptor proteins tested are functionally active, but that E5B9 valency of the TMs and the molecular design of the UniCAR can both influence the antitumoral activity of individual TM and UniCAR-NK cell combinations.

## Discussion

The value of CAR-engineered natural killer cells for the development of safe and effective adoptive cancer immunotherapies is increasingly recognized, with several such approaches now under clinical evaluation [17, 18, 30, 31]. These studies still employ NK cells carrying classical chimeric antigen receptors with an extracellular cell binding domain specific for a single tumor-associated antigen directly linked to transmembrane and intracellular signaling domains. While severe adverse events such as cytokine release syndrome (CRS) or immune effector cell-associated neurotoxicity syndrome (ICANS) that are typically encountered during CAR-T cell therapy have so far not been reported for patients receiving CAR-NK cells [18], on-target/off-tumor toxicity as well as treatment-induced selection of antigen loss variants and ensuing immune escape of cancer cells may still pose considerable risks [5–7].

Here, we achieved tumor-specific targeting of NK cell cytotoxicity utilizing the flexible UniCAR system, which encompasses a CAR directed to the defined E5B9 peptide epitope that is only triggered in the presence of a bispecific adaptor molecule crosslinking target and effector cells [12, 13]. As previously shown for T cells, the UniCAR and the similar RevCAR systems allow to control extent and duration of effector cell activation by adjusting exposure time and dose of the target module. Furthermore, simply by replacing the adaptor molecule of choice, also target cell specificity can be switched without the need to generate new CAR effector cells, thereby addressing critical limitations of current CAR approaches [12, 14, 32]. To thoroughly evaluate the influence of CAR and TM design on functionality of the UniCAR system in NK cells, we generated first- and second-generation UniCARs and a panel of six bispecific adaptors which recognize the tumor-associated ErbB2 antigen overexpressed by many cancers of solid tumor origin. While UniCAR 5B9.z carries transmembrane and intracellular domains of CD3*ζ*, the 5B9.28.z receptor harbors transmembrane and intracellular domains of costimulatory CD28, linked to the intracellular domain of CD3ζ. Similar CARs directly targeting ErbB2 or CD19 were previously shown to facilitate effective cytotoxicity of clinically usable NK-92 cells, which also endogenously express CD3*ζ* and CD28 [16, 21, 33]. Indeed, in the presence of picomolar concentrations of ErbB2-specific target modules both UniCARs efficiently redirected the NK cells to tumor targets expressing high or more moderate levels of ErbB2 on their surface, without affecting ErbB2-negative and otherwise NK-resistant cancer cells. Dependence of cell killing on TM-mediated recognition of ErbB2 was further confirmed by the observed inhibition of cytotoxicity if the TM binding site on target cells was blocked with an ErbB2-specific competitor. Importantly, the extent of tumor-cell killing by a combination of UniCAR 5B9.28.z NK-92 cells and the ErbB2-specific TM 5FE was equivalent to that of clinical-grade NK-92/5.28.z cells that carry a classical ErbB2-specific CAR with CD28 and CD3ζ domains and are currently under evaluation in a phase I clinical trial in glioblastoma patients (clinicaltrials.gov; NCT03383978) [30, 34].

To test the influence of target module design on UniCAR-NK activity, we evaluated six different adaptor molecules which each contain the same ErbB2-specific scFv antibody domain [24], fused to an IgG4-derived Fc domain that facilitated homodimerization of the bispecific proteins in a manner similar to natural IgG molecules. Indeed, approximately 82% of 5FE, 60% of 5FE2 and 66% of 5FE3, as well as 52% of F5E, 51% of F5E2 and 55% of F5E3 molecules were present as homodimers in purified protein fractions. While the reason for the observed differences among 5FE-based TMs is presently unclear, in the case of F5E-based molecules the reduced yield of dimers is likely due to positioning the IgG4 hinge domain at the very N-terminus of the molecules. For UniCAR-T cells, so far much smaller monomeric adaptor molecules were used in which the tumor-targeting antibody domain and E5B9 epitope were directly linked [13, 14, 35]. Due to the short in vivo half-life of such proteins of around 30 to 90 min [19, 36], activation-induced proliferation and cytokine release of UniCAR-T cells can be tightly controlled via TM dosing. Such precautions will most likely not be necessary for UniCAR-NK cells. NK-92-based therapeutics are typically irradiated before infusion into patients as a safety measure to prevent in vivo expansion [16, 37–39]. Prolonged exposure to the effector cells is then achieved by repetitive treatment, which can be discontinued in case of adverse events [30]. Increased safety can also be expected for non-irradiated primary CAR-NK cells, which are more limited in their in vivo expansion potential than adoptively transferred CAR-T cells and so far showed less-pronounced long-term engraftment in cancer patients [18]. Hence, larger Fc-linked, homodimeric target modules appear well-suited for combination with UniCAR-NK cells. Indeed, in vivo half-life of such a prototypic TM protein targeted to GD_2_-positive cancers was almost 40 h in preclinical models [19], bypassing the need for infusion pumps to maintain a suitable blood concentration.

The six TM molecules analyzed all displayed bispecific binding to ErbB2 and UniCARs and selectively triggered UniCAR-NK cell cytotoxicity toward cancer cells expressing high or more moderate levels of the target antigen. As demonstrated in competitive binding experiments, positioning the ErbB2-specific antibody domain either in front of the IgG4 Fc region to separate N-terminal scFv and C-terminal E5B9 peptides or directly linking scFv and E5B9 epitopes at the C-terminus of the molecules had little effect on the interaction with either one of the tested UniCARs for TMs carrying the same number of E5B9 peptides. Nevertheless, binding strength toward 5B9.z and 5B9.28.z UniCARs increased in a similar manner for 5FE and F5E designs if two or three E5B9 sequences were incorporated per monomer. Unexpectedly, this resulted in increased TM-triggered cell killing activity only in the case of NK-92/5B9.z cells, while NK-92/5B9.28.z cells were most active if combined with 5FE and F5E target modules that only contain one E5B9 peptide per monomer. This could be due to distinct higher-order receptor complexes formed at the effector/target cell interface because of different conformational constraints introduced by the CD3ζ and CD28 transmembrane domains, or divergent association of the CARs with endogenous signaling components.

As shown by immunoblot analysis under non-reducing conditions, UniCAR 5B9.z can form covalently linked homodimers and heterodimers with endogenous CD3ζ. These may benefit from TMs with two or more E5B9 sequences through recruitment of additional 5B9.z signaling complexes. UniCAR 5B9.28.z was not expressed as a disulfide-linked dimer. Nevertheless, it may still interact with endogenously expressed CD28 in NK-92 cells as recently described for CAR-T cells [40]. But the signaling potential of putative higher order 5B9.28.z or 5B9.28.z/CD28 complexes may then be disturbed rather than reinforced upon recruitment of additional UniCAR molecules by 5FE2 and 5FE3 or F5E2 and F5E3 TMs. Exposure of ErbB2-negative targets such as K562 leukemia cells to UniCAR-NK cells in the presence of multivalent TMs with two or more E5B9 sequences did not result in enhanced basic NK cell activity, making increased tonic signaling by TM-induced higher order UniCAR complexes unlikely. In the case of classical CARs, binding to more proximal or distal epitopes of a target antigen and the resulting differences in the distance between effector and cancer cell surfaces had a significant impact on CAR activity [41]. In contrast, for ErbB2-specific target modules with identical numbers of E5B9 peptides the cell binding domain either being separated from the E5B9 sequences by an IgG4 Fc domain or a G_4_S linker had no decisive effect on UniCAR-NK activity. This suggests that the tested pairs of adaptor molecules provide a similar degree of flexibility, without the position of the Fc domain negatively affecting formation of the immunological synapse. Consequently, for best activity UniCARs containing a CD3ζ transmembrane domain can be combined with TMs that carry two or more E5B9 peptides, while UniCARs with a CD28 transmembrane domain benefit most from TMs with only one E5B9 motif per monomer. Among the combinations tested in this study, NK-92/5B9.z cells together with TMs 5FE2 or F5E2 displayed both, potent CAR-mediated activity against targets with high or more moderate ErbB2 expression and pronounced CAR-independent natural cytotoxicity (see Supplementary Fig. 4), which may be particularly advantageous for the treatment of tumors with heterogeneous target antigen expression.

Taken together, our data show that tumor-specific targeting of UniCAR-NK cells with multivalent bispecific adaptor molecules results in selective and effective elimination of cancer cells. Evaluating distinct CAR compositions and different target module designs side by side proved highly valuable to identify combinations most promising for further development of UniCAR-NK cells for cancer immunotherapy, and may also be warranted for other adaptor CAR systems currently explored in the field.

## Supplementary Information

Below is the link to the electronic supplementary material.

Supplementary file1 (PDF 6979 kb)
