# Supplementary material for: Multivalent adaptor proteins specifically target NK cells carrying a universal chimeric antigen receptor to ErbB2 (HER2)-expressing cancers
Source: Cancer Immunol Immunother. 2023 Jan 23;72(9):2905–18. doi: 10.1007/s00262-023-03374-x (PMC10412657; doi:10.1007/s00262-023-03374-x)
Supplement: Supplementary file 1 — Supplementary file1 (PDF 6979 kb) [file 262_2023_3374_MOESM1_ESM.pdf]

Supplementary Material

Multivalent adaptor proteins specifically target NK cells carrying a universal chimeric antigen receptor to ErbB2 (HER2)-expressing cancers

Jordi Pfeifer Serrahima, Congcong Zhang, Pranav Oberoi, Malena Bodden,
Jasmin Röder, Claudia Arndt, Anja Feldmann, Anne Kiefer, Maren Prüfer, Ines Kühnel,
Torsten Tonn, Michael Bachmann, Winfried S. Wels

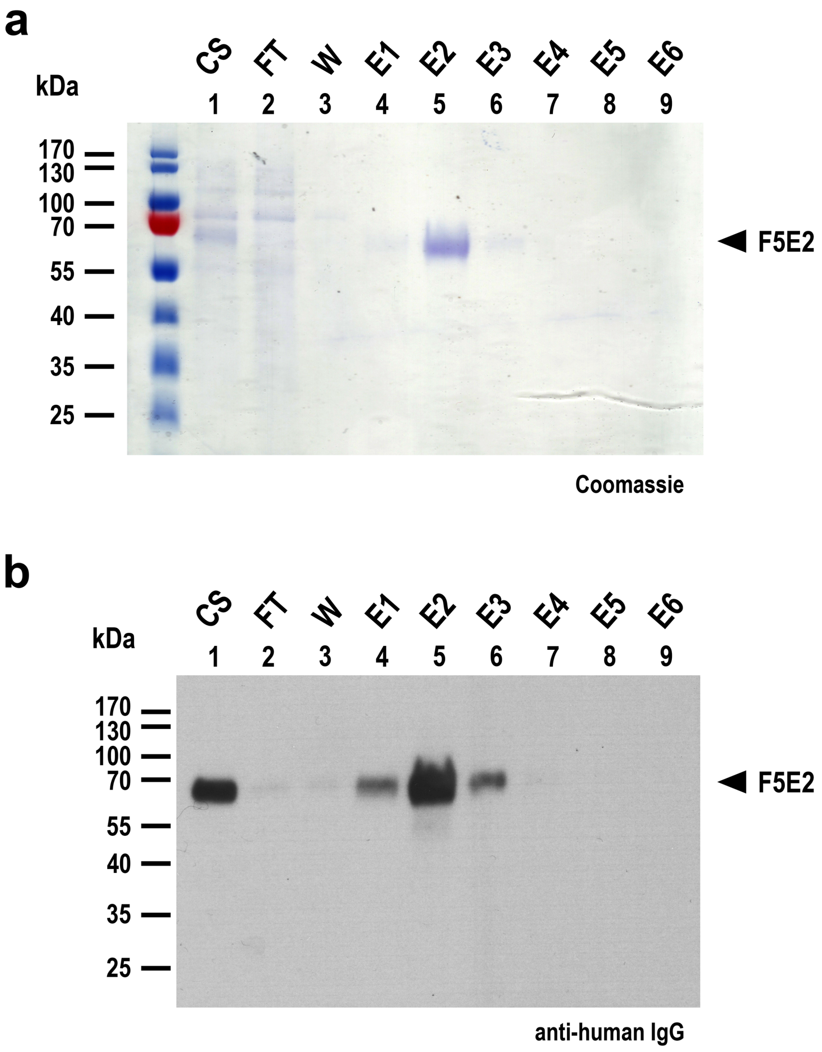

Supplementary Fig. 1 Purification of bispecific target modules. Recombinant TM proteins were expressed as soluble proteins in Expi293F cells and purified from culture supernatants by Protein G affinity chromatography as exemplarily shown for TM F5E2. **a** SDS-PAGE analysis of culture supernatant (CS), flow through (FT), wash fraction (W) and elution fractions (E) 1 to 6 under reducing conditions followed by Coomassie staining. **b** Immunoblot analysis with HRP-conjugated anti-human IgG antibody.

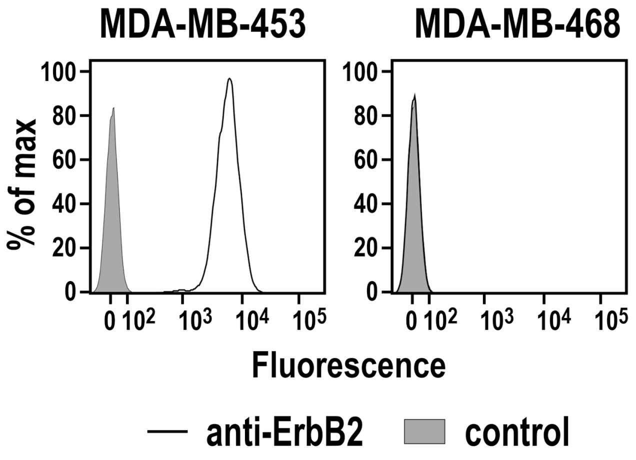

Supplementary Fig. 2 ErbB2 surface expression of MDA-MB-453 and MDA-MB-468 breast carcinoma cells was analyzed by flow cytometry with directly labeled anti-ErbB2 antibody (solid lines). An isotype-matched irrelevant antibody was included as control (filled areas).

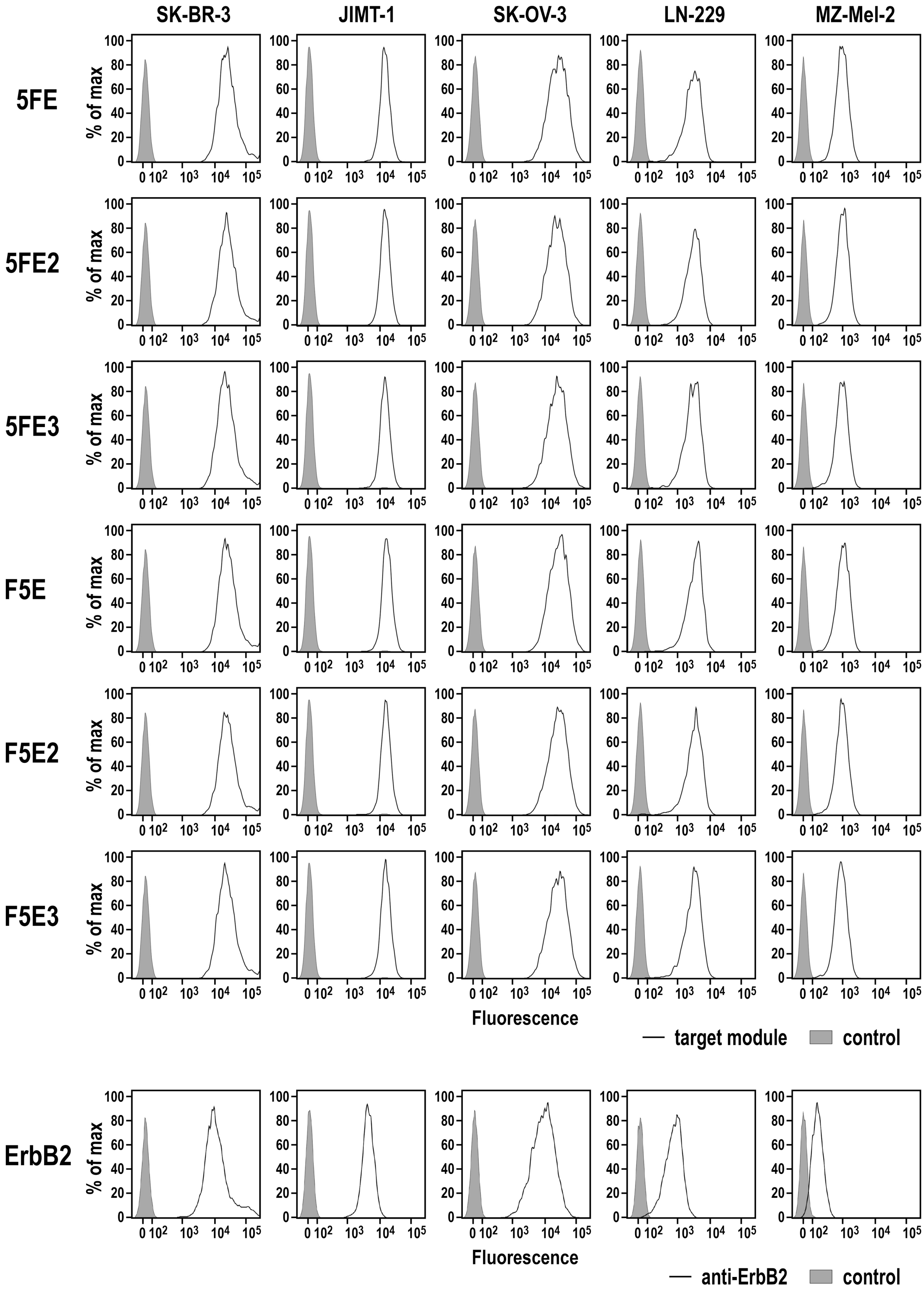

Supplementary Fig. 3 Upper panels: Binding of purified target modules to ErbB2-expressing SK-BR-3 and JIMT-1 breast carcinoma cells, SK-OV-3 ovarian carcinoma cells, LN-229 glioblastoma cells and MZ-Mel-2 melanoma cells was investigated by flow cytometry with anti-human IgG antibody (solid lines). Cells stained with secondary antibody in the absence of a TM served as control (filled areas). Lower panels: For comparison, cells were stained with directly labeled anti-ErbB2 antibody (solid lines) or an isotype-matched control antibody (filled areas).

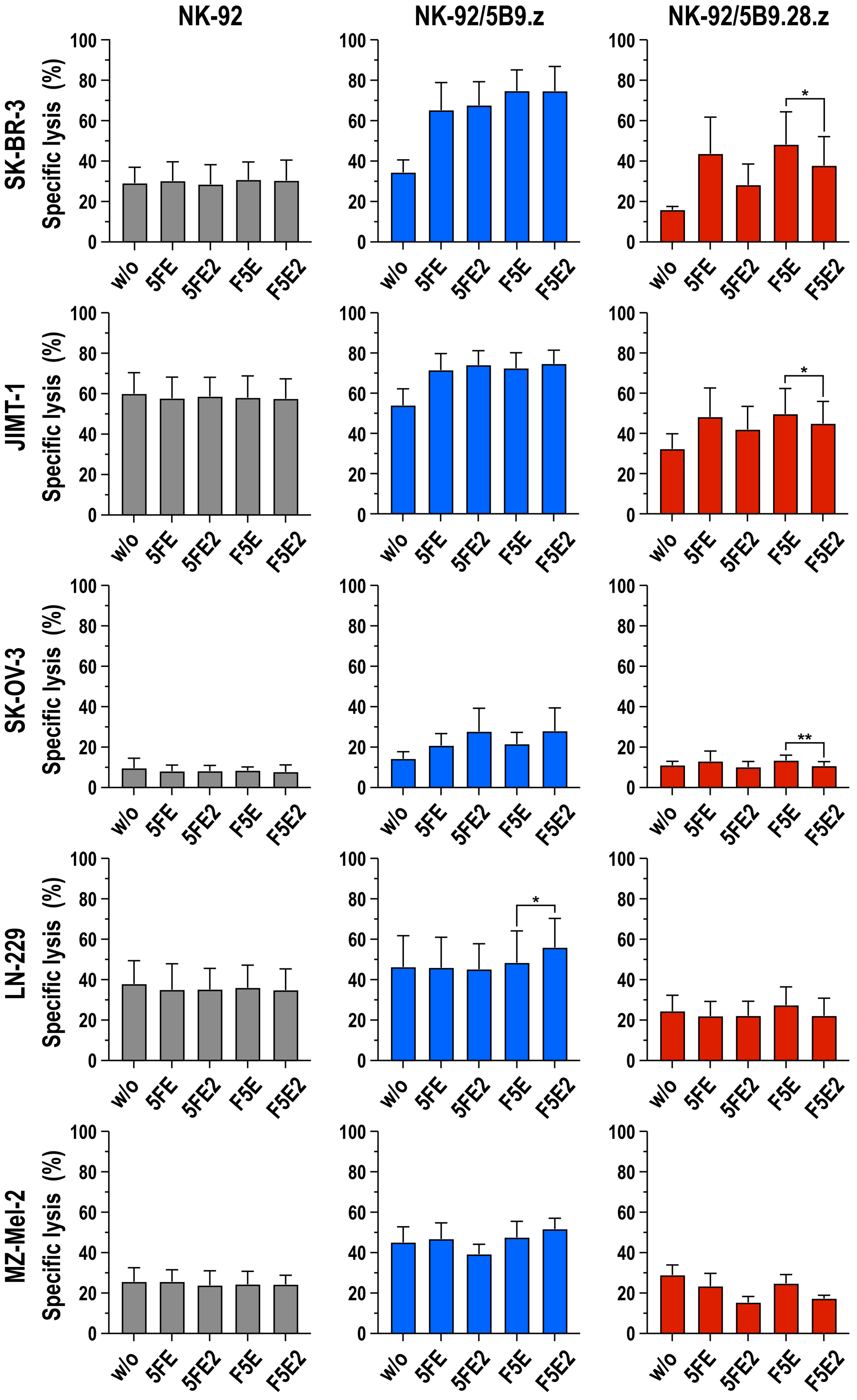

Supplementary Fig. 4 Differential effects of ErbB2-specific target modules on the cytotoxic activity of UniCAR-expressing NK-92/5B9.z (blue bars) and NK-92/5B9.28.z cells (red bars) against SK-BR-3 and JIMT-1 breast carcinoma, SK-OV-3 ovarian carcinoma, LN-229 glioblastoma and MZ-Mel-2 melanoma cells were investigated after co-incubation of effector and target cells in the absence or presence of 0.32 nM of target modules 5FE, 5FE2, F5E or F5E2 at an E/T ratio of 5:1 for 3 hours. Parental NK-92 cells were included for comparison (gray bars).
